# The effect of gender medicine education in GP training: a prospective cohort study

**DOI:** 10.1007/s40037-014-0122-3

**Published:** 2014-06-04

**Authors:** Patrick Dielissen, Petra Verdonk, Magreet Wieringa-de Waard, Ben Bottema, Toine Lagro-Janssen

**Affiliations:** 1 Department of Primary and Community Care, Radboud University Medical Centre, PO Box 9101, 6500 HB Nijmegen, the Netherlands; 2 Department of Medical Humanities, EMGO Institute for Health and Care Research, School of Medical Sciences, VU University Medical Center, Amsterdam, the Netherlands; 3 Academic Medical Center, University of Amsterdam, Amsterdam, the Netherlands; 4 Gender and Women’s Studies, Radboud University Medical Centre, Nijmegen, the Netherlands

**Keywords:** Gender medicine, Medical education, Curriculum development, Evaluation, General practice training

## Abstract

The purpose of this study is to compare the change in general practitioner (GP) trainees’ gender awareness following a modular gender medicine programme or a mainstream gender medicine programme. In 2007, a prospective study was conducted in three cohorts of in total 207 GP trainees who entered GP training in the Netherlands. The outcome measure was the Nijmegen Gender Awareness in Medicine Scale and a 16-item gender knowledge questionnaire. Two gender medicine teaching methods were compared: a modular approach (n = 75) versus a mainstream approach (n = 72). Both strategies were compared with a control cohort (n = 60). Statistical analysis included analysis of variance and t-tests. The overall response rates for the modular, mainstream and control cohort were 78, 72 and 82 %, respectively. There was a significant difference in change in gender knowledge scores between the modular cohort compared with the mainstream and control cohort (*p* = 0.049). There were no statistical differences between the cohorts on gender sensitivity and gender role ideology. At entry and end, female GP trainees demonstrated significantly higher gender awareness than male GP trainees. A modular teaching method is not a more favourable educational method to teach gender medicine in GP training. Female GP trainees are more gender aware, but male GP trainees are not unaware of gender-related issues.

## Background

Appropriate teaching of general practitioners (GPs) is crucial to improve the delivery of gender-specific primary care [1]. Therefore, gender medicine education is nowadays recommended as an integral part of primary care and postgraduate training [1, 2]. Gender medicine education involves the implementation of education about sex- and gender-related processes, reactions and treatments in health care [3]. The World Health Organization supports this gender-based approach in health and illness and has set out specific targets aimed at gender mainstreaming in medical education and health care. Various consensus statements in medical curricula and communication include gender and lay emphasis on training and awareness of gender in health [4, 5]. Doctors are frequently confronted with gender-specific health problems and it is for these reasons that medical schools increasingly take initiatives to provide doctors with appropriate educational curricula on gender medicine [6–10].

Evaluation is vital if we are to answer the question of whether gender medicine education can help to produce doctors who have gender awareness and knowledge. Gender medicine education is still a relatively young scientific domain and little is known about its effectiveness. To date, studies evaluating gender medicine education show positive attitudes of future doctors towards providing women’s health and gender medicine education [8–11]. There is an interest in women’s health and gender issues and the subject is rated as an important one [11–13]. At the same time, conflicting results are reported on the effects of this education, for example in patient management or knowledge scores [6, 7, 14]. Reasons for these conflicting outcomes may be attributed to several limitations of current studies. First, the studies include different educational activities (electives, modules, mandatory) and an explanation of the nature of the educational intervention is not always given [2, 15–18]. This makes it difficult to compare the outcomes and, in fact, may demonstrate a lack of consensus about the best educational approach to teach gender medicine. Second, many educational interventions teaching gender medicine are not in line with current evidence on effective medical education, i.e. targeted, interactive education and more than one intervention preferably extended over time [6, 11, 13, 18]. Third, most of the studies used relatively weak research designs, for example cross-sectional or retrospective evaluations [9, 13, 15, 18, 19]. Last, the validity and reliability of the instruments used are generally not assessed or reported, hence making it difficult to compare and merit the research results [10, 15, 20, 21]. This limits a valid insight into the effectiveness of gender medicine education and warrants the acquisition of more evidence on the effectiveness of gender medicine education.

Educators need to know what strategies are effective when teaching gender medicine. We know that the most effective medical educational methods are the most interactive ones and when more than one intervention occurs. Effectiveness increases in particular if these interventions are extended over time [22, 23]. It would be interesting to know the effect of a mandatory gender medicine programme, which includes the aforementioned educational principles, on GP trainees gender awareness and knowledge. A gender medicine programme with a specific focus on the biopsychosocial aspect of gender would be such an approach to improve the probability of changing GP trainees’ attitude and knowledge. In GP training, a specific focus on gender medicine involves (1) addressing gender issues that are relevant for GPs and frequently seen, (2) having a supervisor with content expertise and (3) educational activities that stimulate GP trainees reflection on their own gender in order to increase awareness of themselves as a woman or a man [24, 25]. Also, GP trainees’ participation should be required because it is a strong force in the acceptance of the subject by students as well as the faculty [26, 27].

Research on gender medicine education has noted that female students benefit more from gender medicine education than male students do and they evaluate programmes better [6, 17, 19]. Reasons for the differences appear to be a result of female students’ greater personal interest in gender-related issues. Also, the topics used in educational programmes may be perceived by male doctors as pertaining more to women’s specific experiences or the perception that gender issues are women’s issues [6, 17]. Furthermore, male students may not be receiving adequate training in gender issues or perceive educational inequality. For example, a prior study showed that male primary care residents had a significantly lower number of women’s health visits per resident year and they had fewer experiences with both acute and preventive women’s health care [19]. Male medical students reported inadequate participation on the obstetrics and gynaecology services, e.g. they were not permitted to perform pelvic examinations by both women patients and by staff [28, 29]. This warrants a more in depth assessment of male and female GP trainees’ gender awareness and level of gender knowledge in order to determine the adequacy of GP training on this subject.

The first purpose of this study is to compare the change in GP trainees’ gender awareness and gender knowledge following a modular gender medicine programme with a mainstream gender medicine programme and a non-systematic gender programme, respectively. The second purpose is to determine whether gender differences in GP trainees gender awareness and knowledge are apparent and persist over the 3-year GP training programme.

## Methods

### Study group

In 2007, 207 GP trainees entered GP training at three institutes in the Netherlands (Nijmegen, AMC Amsterdam and Leiden). All 207 GP trainees were invited and 204 participated voluntarily in the study: 72 in Nijmegen, 72 in Amsterdam and 60 in Leiden. To preserve anonymity, GP trainees were identified using identification numbers. Individual scores from 2007 were matched with those from 2010 to 2011. A key person at each institute held a list with the identification numbers and assured that both the pre- and post-test were completed by the same GP trainee.

### Study design and research intervention

We conducted a prospective cohort study. We used three cohorts over a 3-year period for comparison. The intervention cohorts attended gender medicine education with a different educational approach: a modular approach for the Nijmegen cohort and a mainstream approach for the AMC Amsterdam cohort. A control cohort (Leiden) did not follow any gender medicine education within the existing programme. The three teaching approaches were already in place at the three institutes, i.e. the teaching methods were not randomly allocated.

In the Netherlands, the 3-year GP training is a competency and clinically mainstream based postgraduate curriculum which is similar for all three cohorts. The first and third year are reserved for training in a general practice and the second year is dedicated to rotations in a hospital/emergency room, clinics in a nursing home and a psychiatric outpatient clinic. Throughout their training, GP trainees are supervised by a GP trainer. In addition, GP trainees attend a weekly day release course (10 to 15 GP trainees) at the training institute for theoretical education, clinical and communication skills training and reflection. During these courses, GP trainees receive gender medicine education.

To answer our research question we compared the effect of two teaching methods that address gender medicine education in GP training (Box 1). The modular cohort attended five tutorials of 3 h each spread out over time with explicit gender medicine education from a biopsychosocial perspective and based on effective medical education (interactive, reflective, extended over time). The tutorials focused on gender issues frequently seen by and relevant for GPs such as cardiovascular disease and depression, and were supervised by a GP trainer with content expertise (Table 1) [2]. The mainstream cohort attended traditional courses that, where relevant, included gender medicine information based on a biomedical perspective but without an explicit focus on the different dimensions of gender. The focus was predominantly on medical knowledge and to a lesser extent on the psychosocial context in which both women and men function. The traditional courses were supervised by a GP trainer without specific gender expertise. Both educational approaches aimed to teach GP trainees about gender medicine in GP training. The control cohort attended no systematic educational activities on gender medicine. Box 1 outlines the key elements of the three training programmes.

**Box 1:** Principles and content of gender medicine education

	Modular cohort	Mainstream cohort	Control cohort
Principles for teaching	Biopsychosocial perspective	Biomedical perspective	–
	Knowledge, attitude and skills	Knowledge	–
	Multiple educational activities	Multiple educational activities	–
	GP supervisor with content expertise	GP supervisor	GP supervisor with content expertise
	Extended over time	Extended over time	–
	Encourage reflection	–	–
Content of training	Gender socialization^a^	Gender socialization^a^	Domestic violence^b^
	Gender and doctor-patient communication^a^	Gender in sexually transmitted disease^a^	Sexual abuse^b^
	Gender and mental disorders^b^	Gender in doctor-patient communication^a^	Acute topics in women’s health^b^
	Gender and cardiovascular disease^c^	Gender and depression^b^	
	Gender and intimate partner abuse^c^	Gender and domestic violence^b^	
		Gender and cardiovascular disease^c^	
		Gender in medically unexplained symptoms^c^	

^a ^Year 1; ^ b ^year 2; ^ c ^year 3

**Table 1:** The main factors of the modular gender medicine curriculum in GP training in Nijmegen

Tutorial theme	Main objectives	Teaching methods
1. Gender and socialization	1. Be able to understand the concept of gender	A discourse on the subject (lecture)
	2. Be able to initiate a gender perspective in medical encounters	Group analysis of a video consultation
	3. Awareness of the existence of gender socialization and its implications for health issues	Group reflection on subject with regard to content and process
2. Gender and communication	1. Understanding of the influence of gender in doctor-patient communication	A discourse on the subject (lecture)
	2. Understanding of how gender influences the process of medical decision-making	Role play with simulation patients
	3. Demonstrating gender-sensitive doctor-patient communication	Group reflection on subject with regard to content and process
3. Gender and psychiatric disorders	1. Be able to describe gender differences in depression, anxiety disorders, and substance abuse	A discourse on the subject (a lecture)
	2. Be able to identify gender differences in social expectations with regard to substance abuse	Group reflection on subject with regard to content and process
	3. Be able to recognize male and female presentation and coping in depression and alcohol abuse	Analysis of case reports
4. Gender and cardiovascular diseases/urinary incontinence	1. Be able to understand the gender bias in the care of patients with cardiovascular disease	Pretest to assess gender knowledge
	2. A willingness and ability to minimize the effect of gender bias in cardiovascular disease management	A lecture on gender differences
	3. Be able to describe and recognize the gender differences in presentation and management of urinary incontinence	Group analysis of a ideo consultation
5. Gender and sexual abuse	1. Be able to describe the patterns and common presentations of sexual violence	A discourse on the subject (lecture)
	2. To increase awareness of sexual violence, potential gender prejudices, and consultation skills	Role play with simulation patients
	3. Be able to demonstrate gender-sensitive consultation skills to promote case-finding of sexual abused patients	Group reflection on subject with regard to content and process

**Table 2:** Socio-demographic characteristics of the cohorts at entry

	Modular	Mainstream	Control	*P* ^a^
	N = 72	N = 72	N = 60	
Female (%)	47 (65.3)	55 (76.4)	37 (61.7)	0.160
Age (mean, SD)	29.8 (4.2)	29.5 (3.7)	29.6 (4.3)	0.936
*Self-reported ethnicity (%)*				
Western	64 (88.9)	66 (91.7)	53 (88.3)	0.527
Non-Western	5 (6.9)	2 (2.8)	3 (5.0)	
Unknown	3 (4.2)	4 (5.6)	4 (6.7)	
Hospital working experience (%)	36 (50.0)	40 (55.6)	29 (48.3)	0.824
Out of hospital working experience (%)	9 (12.5)	10 (13.9)	12 (20.0)	
Both	16 (22.2)	10 (13.9)	8 (13.3)	
Other working experience	11 (15.3)	12 (16.7)	11 (18.3)	
*Working experience, years (%)*				0.851
<1 year	24 (33.4)	20 (27.7)	24 (40.0)	
1–3 years	29 (40.3)	42 (58.3)	26 (43.3)	
>3 years	18 (25.0)	10 (14.0)	9 (15.0)	
Unknown	1 (1.3)	0	1 (1.7)	
Former gender education (%)	44 (61.1)	20 (27.8)	26 (43.3)	0.000^b^
No former gender education (%)	28 (38.9)	51 (70.8)	33 (55.0)	
Unknown	0	1 (1.4)	1 (1.7)	

^a^One-way ANOVA (means) or Chi square (percentages)

^b^ *p* < 0.05; comparison statistical significant

### Research instrument

We used the Nijmegen Gender Awareness in Medicine Scale (N-GAMS) to measure gender awareness at entry and at completion of GP training. The N-GAMS was specifically designed for medical education research and its psychometric features have been reported previously [30]. It was used in another sample of GP trainees earlier as well as among medical students [2, 31]. In three subscales, N-GAMS measures the following dimensions of gender awareness: (1) Gender Sensitivity (GS), which focuses on GP trainees’ attitudes towards gender concerns in health care (14 items), (2) Gender Role Ideology Patients (GRI-P), which measures gender stereotyping towards patients (11 items), and (3) Gender Role Ideology Doctors (GRI-D), which measures gender stereotyping towards doctors (7 items). For each subscale, participants indicated their level of agreement with each statement using a 5-point Likert scale (1 = strongly agree, 5 = strongly disagree). A high score on the gender sensitivity scale affirms the consideration of gender in health and illness. High scores on the GRI subscales indicate higher agreement with gender stereotypes about patients or doctors. To assess GP trainees’ knowledge we included 16 questions on gender-specific medical conditions related to or frequently seen in general practice. The participants were requested to rate statements on gender-specific medical conditions as ‘true’ or ‘false’. For example:

1. Cardiovascular disease is the leading cause of death in men as well as in women.
2. Genital discharge is a key symptom of a sexually transmitted infection in men but not in women.
3. Bladder training is effective in women with urge incontinence but not in men.

We used self-declared anonymized information to provide basic socio-demographic information of the GP trainees including age, sex, self-reported ethnicity and previous courses followed on gender medicine. A cover letter explained the aim of our study and indicated that participation was optional.

### Ethical approval

Formal ethical approval for this study was not required by the ethics committee of Radboud University Nijmegen Medical Centre because of the non-invasive character of the questionnaire. The researchers did not have any influence on the curriculum at each GP training institute and the study did not require an intervention at curricular level. GP trainees received education as usual. Also, the NVMO-Ethical Review Board (2010) was not operative at the time of the study.

### Data analysis

We used SPSS version 16 for data analysis. First, we recoded items of the GS subscale that were scored in reverse. We used parametric tests to analyse our data as each subscale consists of 7 or more items [32]. The N-GAMS subscales’ reliability scores were internally consistent. Cronbach’s alpha ranged from 0.68 to 0.91 with the exception of the modular cohort’s baseline score on the GRI-D subscale (α = 0.61).

The analysis consisted of the following:

1. Chi squared tests to examine demographic characteristics (categorical variables) between (1) modular cohort and mainstream cohort and (2) between modular cohort and control cohort.
2. Analysis of variance (ANOVA) to examine demographic characteristics between the cohorts (means).
3. Independent t-tests to examine differences of mean subscale scores between males and females.
4. Dependent t-tests to compare the mean subscale scores at entry and end for each cohort, for males and females.
5. Eta squared to define the proportion of variance associated with or accounted for by the teaching method (effect size). Eta squared varies between 0 and 1, and is interpreted in the usual way, i.e. 0–0.1 is a weak effect, 0.1–0.3 is a modest effect, 0.3–0.5 is a moderate effect and >0.5 is a strong effect.

A *p* value of 0.05 was used as significance level. Non-response bias was explored comparing the results at entry of GP trainees who did and did not complete the second questionnaire. No significant differences in scores were found. Follow-up bias was reduced by using different methods of contact by the key figures (telephone, email, post).

## Results

### Cohort response

The cohort’s response rate to the N-GAMS and gender knowledge questionnaire varied slightly. The overall response rate was 98.5 % (139 females, 65 males) at entry to GP training and 67.6 % (99 females, 39 males) at the end of GP training. A total of 24 GP trainees left GP training prematurely (modular cohort 11, mainstream cohort 10 and control cohort 3).

In the follow-up in 2010–2011, 48 GP trainees of the modular cohort, 45 GP trainees of the mainstream cohort and 47 GP trainees of the control cohort completed the N-GAMS, representing 78.7, 82.5, and 72.6 % of the eligible GP trainees who started the course in 2007. There were no significant differences between the cohorts with regard to gender, age, self-reported ethnicity and working experience at entry (Table 2). At entry, GP trainees in the modular cohort had significantly more gender educational background than their corresponding colleagues.

### Gender awareness and knowledge

Few significant differences were found between the three cohorts with the following exceptions (Table 3). When comparing the three cohorts in one analysis, a significant difference was found among mean scores on gender knowledge but not on gender sensitivity and gender stereotyping (F = 3.087, *df* 2, *p* = 0.049). The effect sizes of the teaching method on the primary outcomes were weak (<0.1).

**Table 3:** Socio-demographic characteristics and N-GAMS subscales scores of three study cohorts

	Modular cohort	N = 48	Mainstream cohort	N = 45	Control cohort	N = 45	p*	Eta squared
	2007	2011	2007	2011	2007	2011		
Age, mean years (SD)	29.6 (4.2)	31.7 (4.5)	29.5 (3.7)	32.4 (3.7)	29.1 (4.3)	32.0 (4.1)	0.840	
Gender, female (%)	32 (66.7)		34 (75.6)		33 (70.2)		0.639	
Western ethnicity, number (%)	44 (91.6)		42 (97.6)		41 (95.5)		0.435	
Working experience, mean years	2.50		2.49		2.38		0.963	
Previous gender education (%)	62.5	100	27.3	77.8	45.7	68.1	0.003^b^	
Gender sensitivity, mean (SD)	3.78 (0.38)	3.98 (0.35)	3.70 (0.36)	3.83 (0.52)	3.65 (0.37)	3.80 (0.32)	0.679	0.006
Mean change in score^a^	0.20^b^		0.13		0.15^b^			
GRI patients, mean (SD)	2.42 (0.59)	2.42 (0.48)	2.04 (0.56)	2.21 (0.63)	2.20 (0.59)	2.45 (0.60)	0.138	0.029
Mean change in score^a^	0.00		0.17		0.25^b^			
GRI doctors, mean (SD)	2.41 (0.42)	2.50 (0.45)	2.19 (0.47)	2.50 (0.73)	2.30 (0.47)	2.50 (0.56)	0.288	0.018
Mean change in score^a^	0.09		0.21^b^		0.20^b^			
Gender knowledge, mean (SD)	10.25 (1.59)	11.64 (1.60)	10.47 (1.84)	10.80 (1.64)	9.82 (1.40)	11.08 (1.69)	0.049^b^	0.043
Mean change in score^a^	1.39^b^		0.33		1.26^b^			

One-way ANOVA (means) or Chi square (percentages); to test whether means between cohorts differ

*GRI* gender role ideology (gender stereotyping)

^a^Dependent Student’s *t* test; to test of whether means within cohort differ

^b^ *p* < 0.05; comparisons significant, otherwise all comparisons non-significant

Regarding the change in gender sensitivity within the cohorts, all three cohorts had a higher, more positive, mean score at the end but the change was significant for the modular and control cohort only (Table 3). The mean change in the modular cohort was 0.20, a significant improvement (T = −3.77; *df* 47; *p* < 0.05). The modular cohort had the highest change in gender sensitivity as well as the highest gender sensitivity score at entry and end. The score of the control cohort increased from 3.65 at entry to 3.80 at the end (T = −4.04; *df* 46; *p* < 0.05).

General practitioner trainees in the mainstream and control cohort had higher scores at the end on the GRI-P and GRI-D subscales, indicating that they held more gender-stereotypical attitudes towards both patients and doctors. GRI-P and GRI-D mean scores at entry and end did not change in the modular cohort. In the mainstream cohort, the mean score on the GRI-D increased significantly from 2.19 to 2.50 (T = −2.47; *df* 44; *p* < 0.05). In the control cohort, the mean change score on the GRI-P as well as the GRI-D increased, reflecting more gender stereotyping towards patients (T = −2.89; *df* 46; *p* < 0.05) and doctors (T = −2.25; *df* 46; *p* < 0.05).

Gender knowledge increased over the course of the GP training for all three cohorts. This improvement was significant in the modular cohort where the score increased from 10.25 to 11.64 (T = −3.84; *df* 47; *p* < 0.05). The same can be said of the control cohort with an increase in score from 9.82 to 11.08 (T = −3.94; *df* 46; *p* < 0.05). The mainstream cohort, however, increased in score from 10.47 to 10.80. This improvement did not reach statistical significance.

### Gender differences

Both genders increased their gender knowledge but the mean gender knowledge scores did not differ significantly between male and female GP trainees. Interestingly, mean scores of male GP trainees’ at entry were lower than those for female GP trainees’ but higher at the end of training (male mean change 1.5 versus female mean change 0.8, *p* = 0.06). In terms of knowledge gain, men seem to benefit more from gender medicine education (Table 4).

**Table 4:** Gender differences on N-GAMS and gender knowledge scores

	Gender sensitivity			GRI patients			GRI doctors			Gender knowledge		
	F	M	*P* ^a^	F	M	*P* ^a^	F	M	*P* ^a^	F	M	*P* ^a^
Entry	3.8	3.6	0.003^b^	2.2	2.4	0.018^b^	2.3	2.3	0.70	10.3	9.9	0.16
End	3.9	3.7	0.002^b^	2.3	2.6	0.001^b^	2.5	2.6	0.17	11.1	11.4	0.25

^a^Independent Student’s t-test; to test whether means between females and males differ

^b^ *p* < 0.05; comparison statistically significant

Female and male GP trainees differed significantly in gender sensitivity mean scores at entry (T = −3.018; *df* 138; *p* = 0.003) and end (T = −3.102; *df* 138; *p* = 0.002), and in GRI-P mean score at entry (T = 2.398; *df* 138; *p* = 0.018) and end (T = 3.551; *df* 138; *p* = 0.01). Both genders had more positive scores on the gender sensitivity subscale and more negative scores on the GRI-P subscale. Female GP trainees were found to have more positive scores on the attitude subscales. There was no overall difference between the female and male GP trainees’ score on the GRI-D subscale.

## Discussion

This study demonstrates that GP trainees following gender medicine education based on a modular teaching method, tailored to effective medical education, are not more gender aware but have gained more gender knowledge during GP training than GP trainees who had other gender medicine education (mainstream, non-systematic). When following a modular programme with a supervisor with content expertise, GP trainees score highest on gender knowledge. The effect of the teaching method on our primary outcomes is very small to absent. We have to bear in mind that GP trainees’ teaching and learning is influenced by many factors in their workplace setting, e.g. role modelling, feedback and reflection. GP trainees following a modular cohort develop a positive change in gender awareness during GP training: a higher gender sensitivity and no more gender-stereotypical attitudes toward doctors and patients. In contrast, the gender awareness of the mainstream and control cohort develops less positively. In both cohorts, gender-stereotypical attitudes become less favourable. This cohort study also shows that for gender sensitivity and gender-stereotypical attitudes towards patients, the attitude scores of female GP trainees are significantly more favourable than those of male GP trainees. Nevertheless, the scores of male GP trainees are not low or negative. Our findings suggest that a modular-based gender medicine programme has no evidence-based preference above other teaching approaches.

Theoretically, the drawback to gender mainstreaming in medical education can be that broadening the focus will lead contradictorily to dilution: separate attention to knowledge, attitude and expertise of gender medicine will fade away making the subject less visible. For example, aspects of gender, integrated in an existing cardiovascular disease course, may be mentioned briefly but touched upon insufficiently for GP trainees to become fully aware of the various dimensions on which gender can influence medicine [1, 33, 34]. Thus, explicit focus on gender medicine, exhibiting features of effective medical education, would show most beneficial effects especially when a supervisor with content expertise is the teacher. This could not be confirmed in our study when comparing the three cohorts directly. The changes within each cohort were more favourable in the modular cohort. Well-informed and motivated staff with regard to gender medicine may have contributed to this effect.

Previous research has reported a relationship between gender and professional attitude towards health care issues as well as between gender and perceived relevance of gender medicine education [6, 7, 13, 17, 19]. Women demonstrate more positive attitude scores and they value gender medicine education higher than men. Despite consistent reports regarding gender differences in the evaluation of gender medicine, our current and other previous findings show that male GPs are not disadvantaged, do not perform poorly and do not exert negative attitudes toward both gender issues and gender medicine education [2, 16].

Study limitations must also be discussed. When considering the effectiveness of gender medicine education in GP training, we have to take into account that the small increase in gender awareness per cohort may also be related to the fact that GP trainees show a growth in professional development including insight into gender issues obtained during GP training. This could be, for example, through role models (GP trainers) and the hidden curriculum; so, becoming a doctor through a process of professionalism that extends beyond the acquisition of biomedical knowledge and clinical skills [35, 36]. Second, more than half of the GP trainees of the control cohort reported to have had some kind of training in gender medicine. A closer analysis of the total curriculum of that cohort reveals some confounders. The curriculum includes gender-related modules, supervised by a GP supervisor with content expertise, on domestic and sexual violence, and acute women’s health. These findings may explain the high percentage of GP trainees in the control cohort that perceived gender medicine through GP training (68.1 %). Unfortunately, we cannot speak of a true control group, as in an experimental study design, considering the high percentage of GP trainees in the control group who had had gender medicine education. Also, it was beyond the possibilities of any of the three GP training institutes to overcome the logistical obstacle implicit in a randomized controlled trial or to control the content of the curriculum during the study period. Last, the observed changes in scores might be significant but they are small as are the effect sizes of the teaching methods. Whether the scores correspond with better gender-sensitive clinical performance is an important question for further research.

In conclusion, we do recommend future gender medicine education in GP training and although our results did not reveal the best educational approach to do so, in our opinion so far a modular one is recommended. Without doubt, the results of our study have several limitations but a modular approach has more favourable outcomes and is best in line with current views of best medical education. Medical education in general favours interactive modular approaches with a specific focus on a subject that extends over time with multiple educational interventions. Further research about the effects of gender medicine in GP training need to also focus on the qualitative aspects of this education. Specifically on how GP trainees’ perceive gender medicine education and in what way it contributes to their professional development as a GP.

## Essentials

- Appropriate teaching of GP trainees is crucial to improve the delivery of gender-specific medicine.
- Gender medicine is recommended as an integral part of postgraduate training.
- Little is known about which teaching method is most effective to teach and learn gender medicine.
- This cohort study does not provide evidence that a modular teaching method is the most effective way to teach gender medicine in GP training.
- Female GP trainees are more gender aware but male GP trainees are not unaware of gender-related issues.
